# Predominance and Diversity of GLRaV-3 in Native Vines of Mediterranean Croatia

**DOI:** 10.3390/plants10010017

**Published:** 2020-12-24

**Authors:** Katarina Hančević, Pasquale Saldarelli, Mate Čarija, Silvija Černi, Goran Zdunić, Ana Mucalo, Tomislav Radić

**Affiliations:** 1 Institute for Adriatic Crops, 21000 Split, Croatia; Mate.Carija@krs.hr (M.Č.); Goran.Zdunic@krs.hr (G.Z.); Ana.Mucalo@krs.hr (A.M.); Tomislav.Radic@krs.hr (T.R.); 2 CNR, Institute for Sustainable Plant Protection, 70126 Bari, Italy; pasquale.saldarelli@ipsp.cnr.it; 3 Department of Biology, Faculty of Science, University of Zagreb, 10000 Zagreb, Croatia; silvija.cerni@biol.pmf.hr

**Keywords:** grapevine viruses, leafroll, autochthonous cultivars, screening, molecular variants

## Abstract

Sixteen grapevine cultivars from Mediterranean Croatia were surveyed for the presence of 10 of the most economically important grapevine viruses. The presence of Grapevine fanleaf virus (GFLV), Arabis mosaic virus (ArMV), Grapevine leafroll associated virus-1, -2, and -3 (GLRaV-1; GLRaV-2 and GLRaV-3), Grapevine virus A (GVA) and B (GVB), Grapevine fleck virus (GFkV), Grapevine rupestris stem pitting associated virus (GRSPaV), and Grapevine Pinot gris virus (GPGV) were tested by reverse transcription polymerase chain reaction (RT-PCR) and enzyme-linked immunosorbent assay (ELISA). All 71 analyzed clones were positive for the presence of one or more viruses. The most abundant one, detected in almost 95% of samples was GLRaV-3. In most of cases it was reported in mixed infections with GVA, GRSPaV, and GPGV. Virus genomes of GLRaV-3 infected vines were further characterized molecularly in order to determine their genetic diversity. Different genomic variants of heat shock 70 protein homologue (HSP70h) were identified by single-strand conformation polymorphism (SSCP) and sequenced. Sequence analysis confirmed their clustering into phylogenetic group I and/or phylogenetic group II. This study emphasizes the wide virus heterogenicity in Mediterranean vines and the predominant presence of GLRaV-3 phylogenetic groups I and II, either individually or in combination.

## 1. Introduction

In comparison to other cultivated crops, grapevines are amongst those infected with the greatest number of viruses. About 80 virus species have so far been identified in this species. The most damaging and widespread grapevine viruses are those causing four major diseases: leafroll disease complex, rugose wood complex, infectious degeneration and decline, and fleck [1].

Grapevine leafroll-associated virus 3 (GLRaV-3), the main causal agent of leafroll disease, is one of the most important virus pathogens worldwide. It is a positive-stranded RNA virus, belonging to the genus Ampelovirus, family Closteroviridae [2]. While symptomless and unrecognized in rootstocks, thus contributing to its spread, important economic losses are reported in *Vitis vinifera* infected with GLRaV-3 [3]. When strongly expressed, GLRaV-3 pathogenicity is evident via leaf downward rolling typical symptoms in red vine variety, delayed ripening, reduced berry sugar content, and a spectrum of disturbed physiological processes, mainly photosynthesis and those related to carbohydrate metabolisms [4].

Grapevine plants are potentially subjected to repeated infections through the mealybugs’ vectors (*Pseudoccocus* spp.; *Planococcus* spp.) and soft scale insects (*Pulvinaria vitis*; [5]) which transmit the GLRaV-3 in semi-persistent manner [3]. Repeated infections together with the error-prone nature of viral RNA-dependent RNA polymerase (RdRps) lead to the accumulation of different GLRaV-3 genetic variants forming complex populations that could easily become a reservoir for events of recombination generating new variants. The knowledge of evolving sequence polymorphisms is essential to fully understand the disease and to ensure the appropriate detection protocol and reliable identification of all significant virus variants. In this way, the estimation of eventual losses and management strategy to prevent vineyard deterioration could be planned and implemented.

Since the first report on GLRaV-3 population structure [6], genetic variability has been brought to the wider attention, so new divergent variants are continuously discovering all around the globe. Phylogenetic classification of GLRaV-3 is mainly based on the nucleotide sequences encoding coat protein (CP), 70 kDa heat shock protein homologue (HSP70h) and RdRp [6,7,8,9]. One of the latest studies based on the genetic diversity of the 3′ terminal region suggests the existence of ten different groups and the majority of genomic variants identified so far could univocally be ascertained to one of the clades proposed by Diaz-Lara et al. [10].

Once infected, symptom expression within each plant depends on virus genotype, environmental conditions and in some minor extant, agrotechnical measures. Some native varieties were found to stand out by their good sanitary status as they were grown in isolation and particularly, not grafted on rootstocks that, although infected, do not show symptoms [11]. Similarly, wild grapevine differs in sanitary status compares to cultivated grapevine [4,12]. With more and more efforts put into preservation of ancient grapevine germplasm, autochthonous varieties represent valuable resources for breeding as well as for diversification in grapevine-derived products. It is expected that some native varieties adequately adapted to a particular environment will adapt easier to the climate changes compared to wide distributed European varieties [13]. In addition to this, comparative status of viral infections of native varieties might give valuable information for the future selection purposes in viticulture.

In this study, 16 most widespread native grapevine varieties from Croatian Adriatic coast were analyzed for the presence and distribution of 10 most important grapevine viruses by both RT-PCR and ELISA. For the most significant pathogen, GLRaV-3, different genomic variants were characterized after their molecular analysis.

## 2. Results

### 2.1. RT-PCR and ELISA Virus Detection

**Figure 1:**
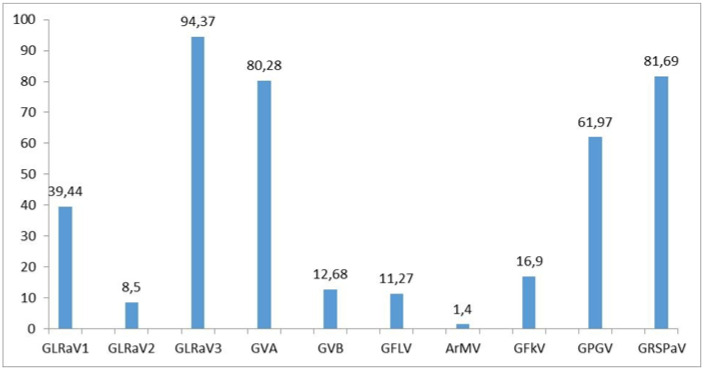
Prevalence of 10 analyzed viruses in 71 samples of autochthonous grapevine varieties at the Croatian Adriatic coast.

In each of 71 analyzed plants, the presence of at least one of the ten tested viruses was confirmed by both molecular and serological assays. The most abundant virus was GLRaV-3, which was detected in 94.37% of plants (Figure 1). Only 4 vines, 3 belonging to cv Pošip crni and one to cv Plavina were found GLRaV-3 free, although infected with other viruses. A high prevalence was also recorded for GRSPaV (81.69%), GVA (80.28%) and GPGV (61.97%), while prevalence of GLRaV-1, GFkV, GVB, GFLV, GLRaV-2, and especially ArMV were much lower (Figure 1).

**Figure 2:**
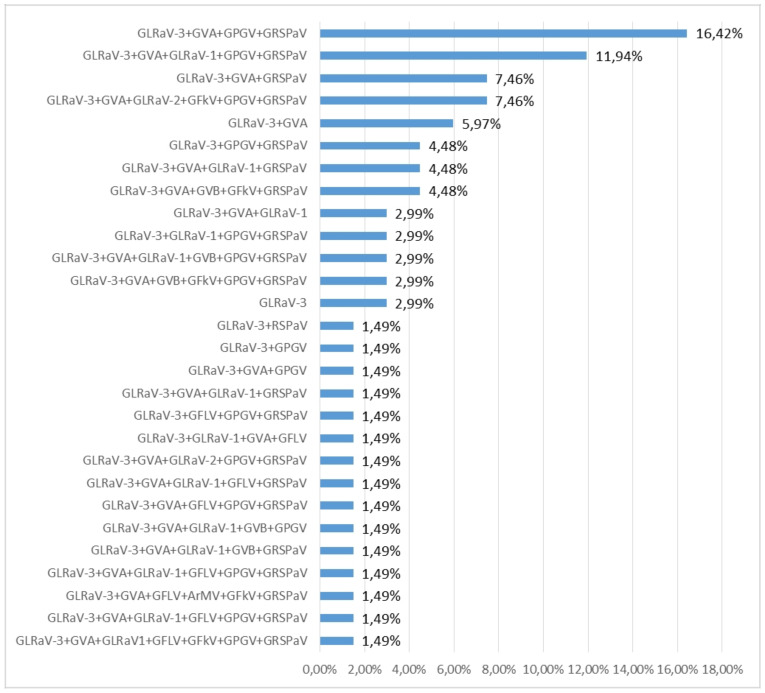
Percentages of different virus combinations infecting tested grapevine samples.

Only two samples of cv Plavac mali were single infected with GLRaV-3, while all other samples had multiple infections. The most common combination (16.42%) comprised GLRaV-3, GVA, GRSPaV, and GPGV (Figure 2).

**Figure 3:**
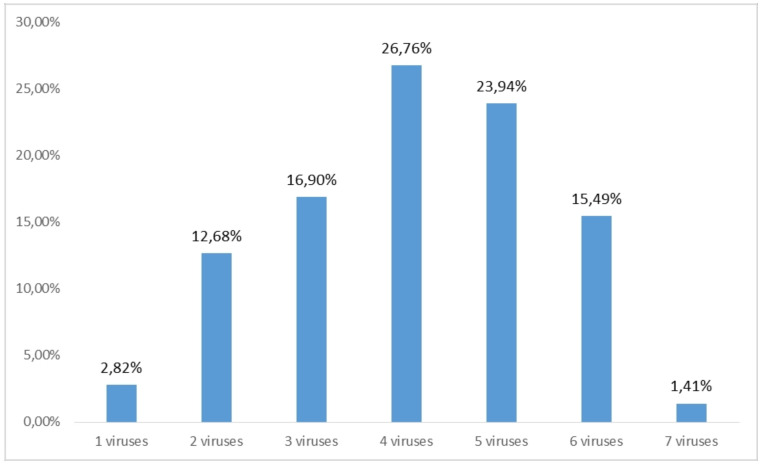
Percentages of single and mixed infections in tested grapevine samples.

**Figure 4:**
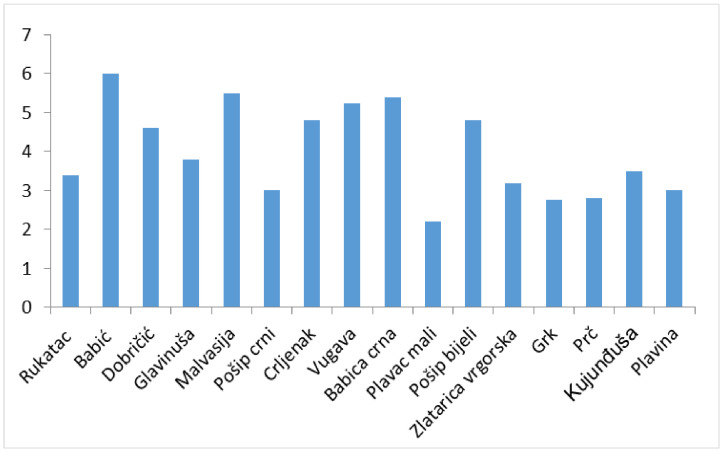
Average number of different viruses detected per grapevine cultivar.

The majority of the samples (26.76%) were infected with four different viruses (Figure 3), while the highest number of different viruses, seven, was detected in one sample of cv Vugava. When looking at the average number of viruses infecting different cultivars, the highest value was recorded in cv Babić and the smallest in cv Plavac mali (Figure 4). A higher sensitivity in diagnostics was accomplished using multiplex PCR in comparison to ELISA test only in the case of three viruses. With GLRaV-2 8.5% of the tested vines were positive using multiplex in comparison to 4.25% gained by using ELISA, in case of ArMV 1.4% of tested vines were positive with multiplex PCR in comparison to 0% using ELISA and finally with GFkV 16.9% of tested vines were positive using multiplex PCR in comparison to 15.49% positive vines using ELISA.

### 2.2. SSCP Analysis and Molecular Diversity of GLRaV-3

**Figure 5:**
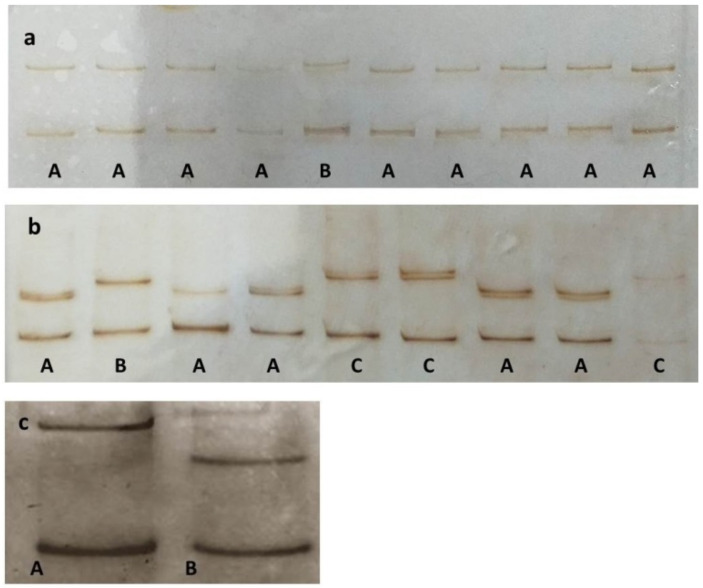
An example of different GLRaV-3-HSP70h haplotypes (marked with letters A, B, C) identified by single-strand conformation polymorphism (SSCP) in monophyletic isolate infecting plants of cv Pošip Bijeli 5 (**a**) and polyphyletic GLRaV-3 isolate infecting plant of cv Vugava 7 (**b**). Different SSCP patterns of variants belonging to group I and II are shown in (**c**).

Thirty-three (33) selected GLRaV-3 positive samples were further analyzed in order to identify and characterize different virus variants present within each isolate. The SSCP analysis of 20 clones per sample whenever possible (in total 586 clones) revealed the existence of more than one genomic variant in tested samples (Figure 5).

**Figure 6:**
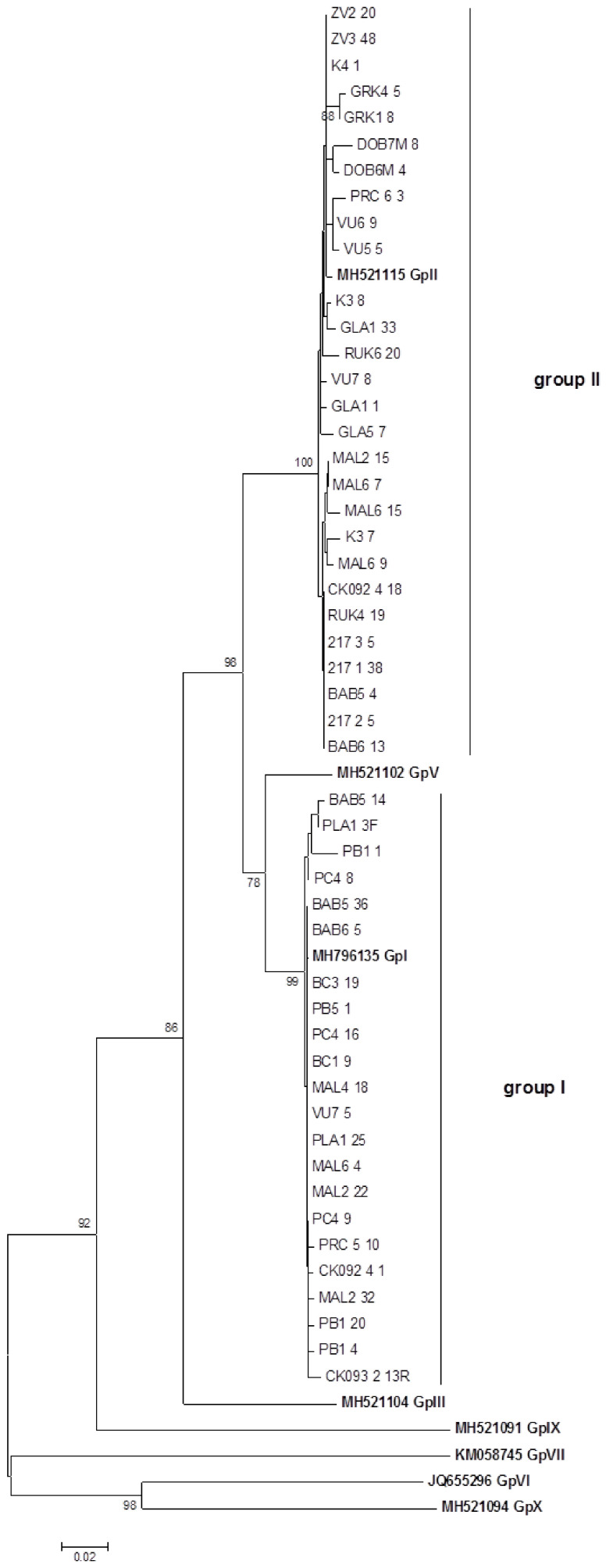
Neighbor-joining phylogenetic tree obtained by the analysis of GLRaV-3 heat shock 70 protein homologue (HSP70h) nucleotide sequences of tested field isolates. Phylogenetic analysis was performed by MEGA 5. The bar represents 0.02 nucleotide substitutions per site. Reference sequences included in the analysis are in bold. Clusters are determined in accordance with the phylogenetic groups published by Diaz-Lara et al. [10].

Different genomic variants were sequenced in a number roughly proportional to its population frequency as suggested by the SSCP results. Not all clones exhibiting different SSCP patterns, showed affiliation to different phylogenic groups (Figure 5). As shown in Figure 5a, two haplotypes were identified. However, sequence analysis confirmed the clustering of both haplotypes into phylogenetic group II. Out of three haplotypes presented on Figure 5b, one clustered into group I (haplotype C), while two clustered into group II (haplotypes A and B). The phylogenetic analysis of all 139 sequenced variants obtained from 33 samples showed clustering into GLRaV-3 phylogenetic groups I and/or II supported by high bootstrap values (≥78%). Genomic variants from 55% of the analyzed samples clustered into phylogenetic group II, while those from 27% of the analyzed samples clustered into phylogenetic group I. Approximately one fifth of samples, 18%, were mixed infected with both variants clustering into both phylogenetic groups I and II. Due to the high number of obtained sequences, only highly represented population sequences of each sample are presented in the phylogenetic tree (Figure 6). The phylogenetic affiliation of each isolate is presented in Table 1.

## 3. Discussion

### 3.1. Virus Distribution

Available results of viral distribution in Croatian vines mostly rely on serological detection tools [14,15,16] so in this study, beside exploiting ELISA, we used RT-PCR for routine detection purposes. Multiplex PCR versus ELISA displayed better sensibility, therefore offering more reliable diagnostic outcomes and accurate assessment of virus presence. Furthermore, the genetic variability of the most widespread virus in autochthonous vines (GLRaV-3) was analyzed by the SSCP method and HSP70h-gene sequencing for the first time.

This research has elucidated GLRaV-3 as the most common virus in native grapevine cultivars from Mediterranean Croatia, highlighting its vast presence in 95% of analyzed vines. GLRaV-3 was mainly detected in mixed infections with GVA, GPGV and RSPaV (Figure 2). Previously, Vončina et al. [17] found a GLRaV-3 high prevalence in the eastern Adriatic vineyards on a limited number of grapevine varieties, mainly using ELISA [14,15,16] and NGS [17].

The damage caused by GLRaV-3 in Croatian vineyards has not been systematically documented, but available data [3] indicate that leafroll diseases could decrease grapevine yield by 15–20% on average, with peaks up to 40%. From the first record on leafroll occurrence in this area, in former Yugoslavia [18], it has been evident that GLRaV-3 is a ubiquitous virus with potentially negative effects on vines. Inspections of viral symptoms in the collection vineyard under study in early autumn showed typical symptoms of leafroll disease in most of the plants, which were not perfectly correlated with the virus composition (Table 1). Leaves redness between green midribs and down rolling were the most obvious symptoms, but for some cultivars (Crljenak kaštelanski and Vugava), severe chlorosis was recorded in the spring, which can be attributed to nepovirus infections. Sporadically, symptoms of leaf mottling and deformation, associated with GPGV, were also recorded. Therefore, these data confirm the harmfulness of GLRaV-3, GLRaV-1, ArMV, GFLV, and GFkV as prescribed by the European Commission directive 2005/43/EC that requires their exemption from certified grapevine plant materials and their inclusion in national certification programs. With no cure for virus-infected plants, only preventive measures are available, consisting of the production and use of clonally selected and sanitized propagation material and vector control.

Beside the confirmed presence of GLRaV-3, as in the case of the other Mediterranean countries [19,20,21,22,23,24], other viruses included in certification programs were not seriously represented in Mediterranean Croatian vines. Fortunately, GLRaV-1 and GFkV were found in lower incidence than some other less harmful viruses (Figure 1). Previous studies based on serological virus detection in Croatian grapevines emphasized GLRaV-1 and GFkV as one of the most frequent viruses [14,16,25] but it referred mainly to continental viticultural regions. Similar findings were reported in Italy [20] leading us to the conclusion that the prevalence of GLRaV-1 in continental viticulture regions is more evident than in coastal Mediterranean part. The incidence of GFkV in our study was also lower (16.9%) than reported for Mediterranean countries [23]

The prevalence of nepoviruses which are considered the most dangerous grapevine viruses was much lower than others (Figure 1). The least represented virus in this study was ArMV with only 1.4%.

After GLRaV-3, the second and the third most abundant viruses in the present study were GRSPaV and GVA associated with the rugose wood complex disease. Confirming data from surveys carried out in the Mediterranean basin [26,27] as well as worldwide [28,29] GRSPaV was found in 81.69% of tested samples. However, although wide-spread, GRSPaV infection has no significant impact on growth and yields and potentially may be even beneficial to the grapevine host [30]. GVA persistently has a high prevalence in Mediterranean countries [23], confirmed here by our study.

GPGV, an emerging virus that is not yet included into viticulture certification programs in Europe, appears to be widely distributed in Croatia. RT-PCR detection revealed its high occurrence in Croatian autochthonous vine cultivars (Figure 1). According to the newest data from European Plant Protection Organization, GPGV is nowadays present worldwide in Asia, Africa, North America, South America, Europe, and Australia. The virus is widely spread in the neighboring Slovenia [31], as in one survey 40 out of 42 symptomatic vines were infected. From 2012 onward, it has been detected in most countries of the Mediterranean basin [3,32,33], United States [34], Germany [35], Canada [28], Lebanon and Middle East [36], China [37] and South Korea [38] and its first report in Croatia comes from Bertazzon et al. [39]. The frequency of GPGV in our study was 61.97% (Figure 1), infecting all cultivars except Prč, which is significantly increased than previously reported [17]. As the analyzed cultivars belong to a germplasm collection and are supposedly not subjected to frequent agronomical (i.e., grafting, propagation) activities, this wide GPGV prevalence is likely due to the presence of a vector, previously showed to be the mite Colomerus vitis [40].

Significant differences in virus distribution were found among cultivars. These may be due to their original vine-growing area, exposure to infection, and/or to their specific susceptibility to different viruses. Plavac mali is the main local cultivar of Mediterranean Croatia, representing 9.1% (1697.3 ha) of the total vineyard acreages in the Country, and considered the second rated cultivar [41]. This cultivar was the only one for which we found replicates infected with solely GLRaV-3 and, in general, it hosted the smallest number of viruses. The reason likely relies on small populations of local varieties that are traditionally propagated in relatively isolated vine-growing areas at Croatian islands like Vis, which allowed the preservation of a good sanitary status (Figure 4). The traditional maintenance of vineyards at Adriatic islands is still characterized by a low intensity of the introduction of propagation materials; some of early rootstocks introduced at the beginning of 20th century are still in use for vineyard establishing. A similar sanitary status was found in old cultivars originated from the islands of Korčula (Grk) and Hvar (Prč; Figure 4) that were infected by the lowest number of viruses. However, the presence of many different viruses has previously been serologically detected in Plavac mali, [16] indicating that the extent of infections does not depend on specific resistance traits. Eight out of 16 cultivars analyzed, originated from islands located along the coast. In comparison to grapevines originated from coastal Adriatic their sanitary status was more superior (average 3.6 viruses per plant vs 4.3 in coastal sites). Babica crna and Babić, the cultivars with the biggest number of viruses, are located in coastal Mediterranean Croatia (Figure 4) with a long tradition of grapevine cultivation

Not only were the differences in viral distribution between cultivars found, but occasionally also between clones of the same cultivar. Further surveys should be conducted to confirm whether some cultivars such as Plavac mali, Grk, or Prč are generally characterized with the lower susceptibility to certain viruses by taking into consideration their complete area of distribution. There are some findings that native varieties stand out for their good sanitary status [11] similar as wild grapevine [12], so the comparative status of viral infections of autochthonous varieties might give valuable information for future selection purposes in viticulture.

No plants were found without viruses, so the deteriorated sanitary status of valuable Croatian cultivars calls for a strategy to ensure virus-free planting material.

### 3.2. Study of GLRaV-3 Natural Population

Due to the highest prevalence, GLRaV-3 was further explored in order to identify and molecularly characterize the different genetic variants present in the tested samples. The obtained sequences clustered into two (groups I and II) out of the ten phylogenetic groups already described [10]. Since this virus was mostly accompanied with other viruses within the analyzed plants, and distributed in different cultivars, the typical expression of symptoms of these variants was difficult to interpret in the field. Combining different methods and genetic regions for variant typing, combination of group I and II in the local grapevines was also reported in Portugal [42] and China [43], with the prevalence of group I in both cases. Along with the variants from other groups, a combination of variants from groups I and II was found in Napa Valley [44]. In 73% of our tested samples a clear predominance of group II variant was detected either alone or in coinfection with variants from group I. Among these, 55% were pure II isolates. On the contrary, only 27% of samples were found to belong to group I. In most of the countries, variants from groups I and II are accompanied with variants from other phylogenetic groups but based on the number of sequences deposited in GenBank, one could conclude that the predominant GLRaV-3 genotype is genotype from group I followed by group II and group III. However, the predominance of variants from group II reported in our study has been described in Spain [45], Algeria [8] and South Africa [46] as well.

Looking at the distribution of individual viruses in the studied germplasm collection and taking into consideration the mode of their spreading, we can speculate about horizontal virus transmission happened at some point. At the present time, the visual inspection of known viral vectors gave negative results and the collection vineyard is regularly treated against the insect vectors or nematode. Therefore, the correlation of the presented results with the natural incidence of viral infections and their molecular characteristics present in the field cultivars should be explored more cautiously.

## 4. Materials and Methods

### 4.1. Viral Source

The samples were collected from a germplasm collection vineyard located at the Institute for Adriatic Crops in Split, Croatia. The collection was established during the long-term identification and conservation program of old native grapevine varieties, encompassing all main autochthonous cultivars from Mediterranean Croatia collected along the coastal area, more than 350 km long. We selected 16 cultivars belonging to the eastern Adriatic autochthonous set of grapevine cultivars: Babica, Babić, Crljenak, Dobričić, Glavinuša, Grk, Kujunđuša, Malvasia dubrovačka, Plavac mali, Plavina, Pošip bijeli, Pošip crni, Prč, Rukatac, Vugava, and Zlatarica vrgorska. Whenever possible, 5 clone of each cultivar, 71 samples in total, were tested for the presence of ten major grapevine viruses both serologically and molecularly. Both reactions were performed on dormant canes collected in the late winter. Fresh tissue was grinded in liquid nitrogen and proceed further for the screening of Grapevine fanleaf virus (GFKV), Arabis mosaic virus (ArMV), Grapevine leafroll associated virus-1, -2, and -3 (GLRaV-1; GLRaV-2, and GLRaV-3), Grapevine virus A, (GVA) and B (GVB), Grapevine fleck virus (GFkV), Grapevine rupestris stem pitting associated virus (GRSPaV) and Grapevine Pinot gris virus (GPGV). Selected samples that tested positive for GLRaV-3 were further analyzed based on the nucleotide sequence of HSP70h gene.

### 4.2. ELISA Virus Detection

To gain a preliminary insight into the sanitary status of cultivars in the experiment, all 71 samples were tested by available DAS-ELISA (Double antibody sandwich-ELISA) diagnostic tests [47] for: ArMV, GFLV, GLRaV-1, GLRaV-2, GLRaV-3, GVA, GVB, and GFkV (Agritest, Italy). GRSPaV and GPGV were omitted from DAS-ELISA testing due to the unavailability of reliable serological reagents.

After grinding in liquid nitrogen, samples were diluted in 2 mL of extraction buffer, prepared according to the manufacturer instructions. The samples were incubated with capture antibody at 4 °C overnight, followed by the incubation with conjugate antibody next day, for 2 h on 37 °C. Two hours after adding substrate p-nitrophenylphosphate, asorbance was recorded at 405 nm using an automatic microplate reader.

### 4.3. RNA Extractions, cDNA Synthesis and PCR Virus Detection

The total RNA was extracted from 250 mg of cortical scrapings using RNeasy Plant Mini Kit (Qiagen, Hilden, Germany) applying an improved RNA extraction procedure as described by MacKenzie et al. [48]. Reverse transcription [49] was performed using 200 units of MMLV reverse transcriptase (Invitrogen, Waltham, MA, USA), 100 units of RNase inhibitor (Invitrogen, USA), 0.5 mM dNTPs and 2.5 µM random nonamers (Sigma Aldrich, St Louis, MO, USA) in the reaction mixture of 22 µL that contained 12 µL of extracted RNA. The reaction mixture was incubated for 10 min at 25 °C and 60 min at 37 °C followed by 15 min at 70 °C.

The detection of relevant grapevine viruses associated with leafroll (GLRaV-1, -2, and -3), rugose wood (GVA and GVB), infectious degeneration (GFLV and ArMV), and GFkV, was performed by multiplex PCR as reported by Gambino and Gribaudo [49], whereas the detection of GRSPaV was performed as individual reaction using primers described by Meng et al. [50]. In both cases the cycling conditions were initial denaturation at 94° C for 2 min, followed by 35 amplification cycles (30 s at 94 °C, 60 s at 55 °C and 90 s at 72 °C), and final extension of 10 min at 72 °C. The presence of GPGV was determined by separate PCR reaction as described by Saldarelli et al. [51] using specific primer pairs [52]. As indicators of RNA quality and RT-PCR effectiveness, primes for Vitis 18S rRNA were used. Reaction products were analyzed by agarose gel electrophoresis ascertaining the following amplicon sizes: 18S rRNA (844 bp), GPGV (588 bp) GLRaV-2 (543 bp), GVB (460 bp), ArMV (402 bp), GLRaV-3 (336 bp), GVA (272 bp), GLRAV-1 (232 bp), GFkV (179 bp), GRSPaV (155 bp) and GFLV (118 bp.)

### 4.4. Cloning and Single Stranded Conformational Polymorphism Analysis

At least two plants of each of the 16 GLRaV-3-positive cultivars, 33 samples in total, were selected for further molecular characterization. A fragment (545 bp) of the HSP70h gene was amplified using LC1F and LC2R primer pairs [6]. The PCR conditions were as reported by Turturo et al. [6] except for the final extension, which in our case was prolonged to 15 min in order to increase further cloning efficiency. The amplified products were purified using the Wizard Genomic DNA Purification Kit (Promega, Madison, WI, USA). To separate different genomic variants presumably present in each isolate, amplicons were cloned in the pGEM-T Easy Vector Sistem I (Promega, Madison, WI, USA) and ligation mixtures were transformed in Escherichia coli JM109-High-Efficiency Competence cells (Promega, Madison, WI, USA) according to the manufacturer’s instructions. The presence of the insert was confirmed by PCR using the same primers and PCR conditions as mentioned above. To identify different variants and determine their population frequency, whenever possible, 20 transformed colonies per sample were randomly selected and analyzed by single-stranded conformational polymorphism (SSCP) analysis. Aliquots of the amplified products (2 μL) were denatured and separated by native polyacrylamide gel electrophoresis as described by Černi et al. [53]. Different SSCP profiles were visualized after silver staining [54]. DNA fragments displaying different SSCP patterns were considered diverse genomic variants and were selected for sequencing.

### 4.5. Nucleotide Sequence Analysis

Selected amplicons were sequenced in both directions by Macrogen Europe (Amsterdam, Netherland) using primer pair LC1F/LC2R. Representative sequences of each cultivar were deposited in GenBank under the accession numbers: MW316065-MW316084 Reference sequences from different GLRaV-3 phylogenetic groups were selected from Diaz-Lara et al. [10] and retrieved from the GenBank: MH796135 (GpI), MH521115 (GpII), MH521104 (GpIII), MH521102 (GpV), JQ655296 (GpVI), KM058745 (GpVII), MH521091 (GpIX) and MH521094 (GpX). Sequences were aligned using ClustalX 2.1 [55] and analyzed by MEGA version 5 [56] using the neighbor-joining method and applying the Tamura-Nei evolutionary model. The tree topology was evaluated by bootstrap analysis based on 1000 repetitions.

## 5. Conclusions

These are the first results on screening of 10 different viruses in Croatian East Adriatic native grapevines by applying RT-PCR diagnostic tools and on investigating GLRaV-3 genetic variability using SSCP method in Croatia. The study confirmed the dominance of GLRaV-3 and revealed its relatively low genetic variability. The results contribute to the understanding of the global distribution of the virus, the biological significance of coinfections and the potential dangers for the wider region in case of their potential spreading.

## Figures and Tables

**Table 1:** The vine-growing area of grapevine cultivars, the genetic affiliation of GLRaV-3 isolates found, co-occurrence of other viruses, and the presence of leafroll disease symptoms.

Cultivar	Vine-Growing Area	Isolate Name		GLRaV-3 Phylogenetic Group	Co-Occurrence of Other Viruses	Leafroll Symptoms Presence
Babica crna	Kaštela	BC1		I	GVA,GVB,GFkV,GRSPaV	−
Babica crna	Kaštela	BC3		I	GVA,GVB,GFkV,GPGV,GRSPaV	−
Babić	Primošten	Bab5		I+II	GLRaV-2,GVA,GFkV,GPGV,GRSPaV	+
Babić	Primošten	Bab6		I+II	GLRaV-2,GVA,GFkV,GPGV,GRSPaV	+
Crljenak kaštelanski	Kaštela	CK_092/4	CK_093/2	I+II	GVA,GFLV,ArMV,GFkV,GRSPaV	+
Crljenak kaštelanski	Kaštela			I	GVA,GPGV,GRSPaV	+
Dobričić	Šolta	Dob6		II	GLRaV-1,GVA,GRSPaV	+
Dobričić	Šolta	Dob7		II	GLRaV-1,GVA,GRSPaV	+
Glavinuša	Kaštela	Gla1		II	GVA,GPGV,GRSPaV	+
Glavinuša	Kaštela	Gla5		II	GVA,GPGV,GRSPaV	+
Grk	Korčula	Grk1		II	GRSPaV	+
Grk	Korčula	Grk4		II	GPGV,GRSPaV	+
Kujunduša	Imotski	K3		II	GLRaV-2,GVA,GRSPaV	+
Kujunđuša	Imotski	K4		II	GVA,GRSPaV	+
Malvasia dubr.	Konavle	Mal2		I+II	GLRaV-1,GVA,GVB,GPGV,GRSPaV	+
Malvasia dubr.	Konavle	Mal4		I	GLRaV-1,GVA,GVB,GRSPaV	+
Malvasia dubr.	Konavle	Mal6		I+II	GLRaV-1,GVA,GVB,GPGV,GRSPaV	+
Plavac mali	Vis	217/1		II		−
Plavac mali	Vis	217/2		II		−
Plavac mali	Vis	217/3		II	GPGV	−
Plavina	Drniš	Pla1		I	GVA,GPGV,GRSPaV	+
Pošip bijeli	Korčula	PB1		I	GLRaV-1,GVA,GPGV,GRSPaV	−
Pošip bijeli	Korčula	PB5		I	GLRaV-1,GVA,GPGV,GRSPaV	−
Pošip crni	Korčula	PC4		I	GLRaV-1,GPGV,GRSPaV	−
Prč	Hvar	Prc5		I	GLRaV-1,GVA	−
Prč	Hvar	Prc6		II	GVA,GRSPaV	−
Rukatac	Korčula	Ruk4		II	GVA	+
Rukatac	Korčula	Ruk6		II	GLRaV-1,GVA,GRSPaV	+
Vugava	Vis	Vu5		II	GLRaV-1,GVA,GFLV	+
Vugava	Vis	Vu6		II	GLRaV-1,GVA,GFLV,GRSPaV	+
Vugava	Vis	Vu7		I+II	GLRaV1,GVA,GFLV,GFkV,GPGV,GRSPaV	+
Zlatarica vrgorska	Vrgorac	ZV2		II	GVA,GPGV,GRSPaV	−
Zlatarica vrgorska	Vrgorac	ZV3		II	GVA,GRSPaV	−

+ indicates the presence of symptoms that are associated with leafroll disease; − the absence of any visible symptoms.

## Data Availability

Representative sequences were deposited in GenBank under the accession numbers: MW316065-MW316084.
